# RTS,S: the first malaria vaccine

**DOI:** 10.1172/JCI156588

**Published:** 2022-01-04

**Authors:** Fidel Zavala

**Affiliations:** Malaria Research Institute, Department of Molecular Microbiology and Immunology, Johns Hopkins Bloomberg School of Public Health, Baltimore, Maryland, USA.

## The long road to vaccine development

**Figure 1:**
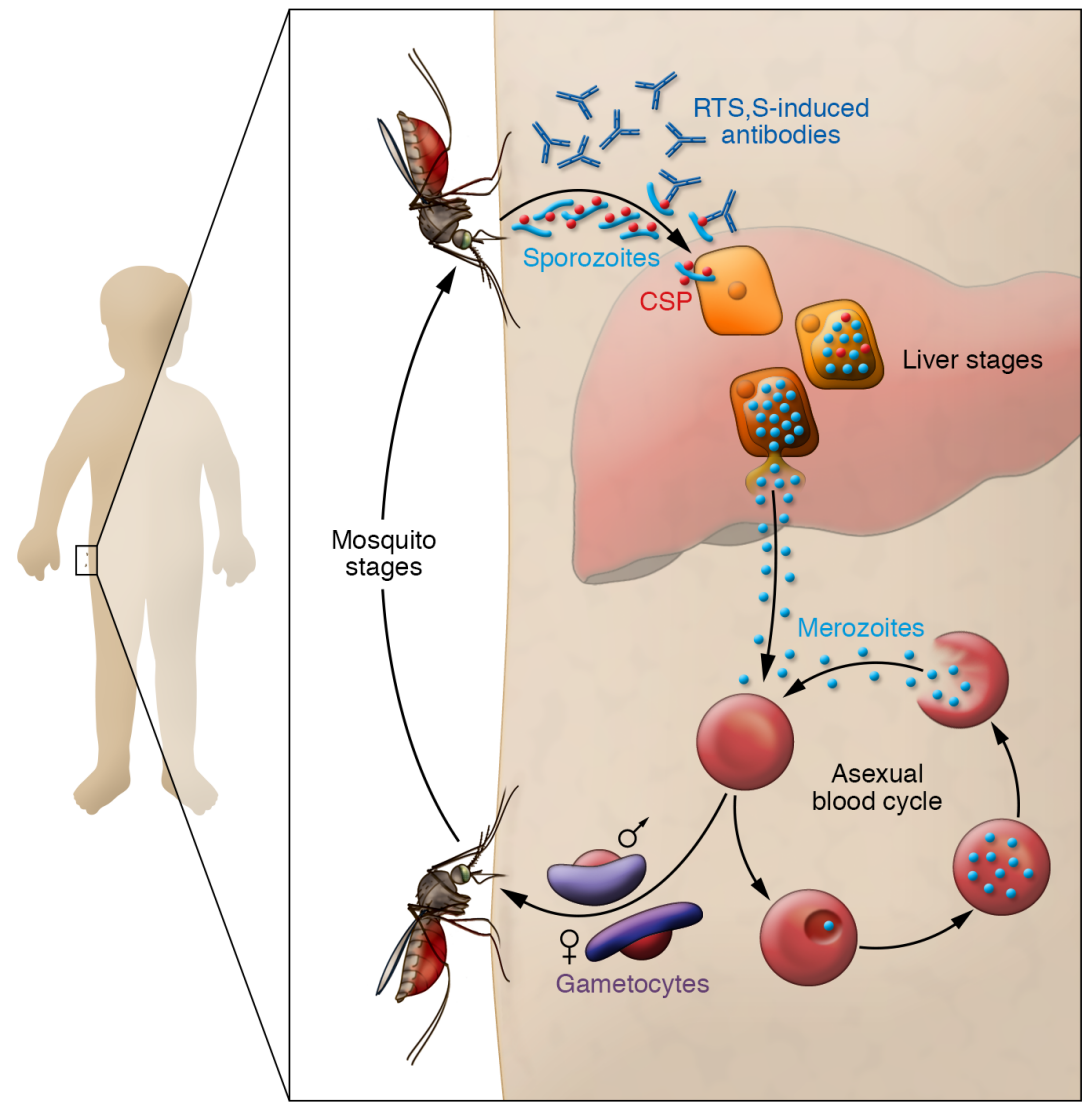
Impact of RTS,S vaccine on malaria infection and transmission. Vaccination with RTS,S induces antibodies against circumsporozoite protein (CSP), which is expressed by sporozoites, the infective form of Plasmodium that mosquitos transmit. During infection in unvaccinated individuals, sporozoites travel to the liver, where they move through hepatocytes and differentiate to hepatic merozoites. CSP is expressed in the early liver stages, but not by liver stage merozoites. Antibodies to CSP following RTS,S vaccination immobilize the sporozoites, thereby preventing infection of hepatocytes. RTS,S-induced protection from infection and severe disease wanes over time and correlates with the level of anti-CSP antibodies. RTS,S-induced immune responses do not interfere with the infectivity of Plasmodium gametocytes to mosquitoes. Even following vaccination, most children will carry parasites that will infect mosquitoes; thus, transmission in the population will remain unchanged. Image adapted from Raphemot et al. (19).

After more than four decades of basic research and clinical trials, the World Health Organization (WHO) has recommended the malaria vaccine RTS,S for widespread use among children living in malaria endemic areas. Pioneering studies using rodent malaria models directed by Ruth S. Nussenzweig at the New York University School of Medicine demonstrated in the late 1960s that immunization with attenuated sporozoites — the infective stage of *Plasmodium* — induces immune responses that protect against parasite infection (1). These studies also identified the circumsporozoite protein (CSP), the sporozoite-specific molecule recognized by the protective immune responses that is the antigen incorporated in the RTS,S vaccine (2). The CSP is expressed on the surface of sporozoites of different *Plasmodium* species and contains a central domain of tandem repeats that represent approximately 30% of the entire sequence. Extensive experimental evidence indicates that binding of antibodies to these repeats immobilizes the sporozoites, preventing infection of hepatocytes, an obligatory stage of this infection (Figure 1). The RTS,S vaccine is a hepatitis B virus–like particle that contains a genetically fused portion of the repeat domain and the C-terminal region of the *P*. *falciparum* CSP (3).

## Clinical data for RTS,S

The first successful human trial demonstrating protection against infection by *P*. *falciparum* sporozoites was conducted in 1996 at the Walter Reed Army Institute of Research using RTS,S developed by Glaxo Smith Kline (4). Several phase II and III vaccine trials were conducted in endemic areas in the last 15 years, and the results consistently indicated that immunization of children 6 to 12 weeks and 5 to 7 months old induces a protective immunity that neutralizes sporozoite infection or attenuates the clinical severity of the infection. An extensive phase III trial that included different endemic areas of Africa indicated that the efficacy against clinical malaria, a few weeks after the last immunization, begins at 74% in children aged 5 to 17 and decreases to 28% and 9% after 1 and 5 years, respectively. In children aged 6 to 12 weeks, the efficacy was estimated to begin at 63% and waned to 11% and 3% after 1 and 5 years, respectively (5). The protective effect of this vaccine is short-lived, and it appears to depend on the intensity of transmission in different endemic areas. This decreased efficacy correlates with reduced levels of anti-CSP antibodies, indicating that protection depends on sustained high levels of circulating antibodies (6). There is only limited information on vaccination of adults. In The Gambia, RTS,S immunization of adults induced short-lived protection from infection on 34% of vaccinees (7), while no significant protection was observed in Kenya (8).

The implementation of RTS,S vaccination programs is a positive first step and according to the WHO it could reduce severe disease in 30% of vaccinated children (9). However, as this vaccine does not provide extensive sterile immunity, and RTS,S-induced immune responses do not interfere with the infectivity of gametocytes (the transmission stages of *Plasmodium*), most children and adults will carry parasites that will infect mosquitoes. Thus, transmission will remain unchanged, ensuring continuous endemicity.

## Next-generation malaria vaccines on the horizon

There is a consensus that major improvements are necessary to develop a vaccine that is likely to have a greater epidemiological impact in endemic areas. Since the development of RTS,S in the late 1980s, continuing research has greatly increased our understanding of the protective immune mechanisms that neutralize parasite infectivity. This research has also yielded a better characterization of factors influencing vaccine-induced immune responses. In fact, new vaccine candidates have been developed that consist of antigenic domains similar to RTS,S expressed in different platforms such as nanoparticles, mRNA, and others. Recently, human trials using a nanoparticle, R21, were conducted with children in Burkina Faso and the initial results indicate that 1 year after 3 immunizations, this vaccine conferred a 77% protection from severe disease (10). New R21 trials in areas with different transmission intensities should provide comprehensive information on the efficacy of this vaccine compared to RTS,S. Another vaccine candidate, attenuated *P*. *falciparum* sporozoites, was also evaluated in adults living in Mali, and the estimated protective efficacy was 29% by proportional analysis (11). A recent trial of this attenuated sporozoite vaccine in Kenya failed to demonstrate significant efficacy in 5- to 12-month-old children (12).

Considerable advances have been achieved regarding the structure and fine specificity of anti-CSP protective antibodies. Recent biophysical studies have characterized the binding properties of protective antibodies, and crystallography studies have defined the precise conformation of the CSP epitopes recognized by these antibodies (13). Importantly, studies with protective human monoclonal antibodies obtained from individuals immunized with sporozoites have identified unique antigenic moieties within the repeat domain of the CSP, which are recognized by protective antibodies but are not included in the RTS,S vaccine (14–16). Thus, there are good reasons to expect that a new generation of structure-based vaccines containing additional antigenic moieties expressed in platforms with enhanced immunogenicity will improve the quality of the antibody response and its efficacy against sporozoite infection. Another aspect that has yet to be systematically explored is the development of vaccines designed to induce CD8^+^ T cell immunity in humans. While the protective effect of CD8^+^ T cells that recognize parasite epitopes presented by infected hepatocytes is firmly established in animal models, translating this knowledge to develop new human vaccines is still a major challenge, due to severe methodological limitations.

## Ongoing challenges

The protracted development and moderate efficacy of the RTS,S vaccine, in sharp contrast with the swift development of highly protective COVID-19 vaccines, underline the major differences that exist between immunity against viruses and malaria sporozoites. Vaccine-induced antiviral immune responses act by neutralizing the virus at the start of the infection and continue to exert this effect on viral particles released from infected cells, so the immune pressure is constant during the infection. It may be strengthened by secondary antibody responses that are likely to occur during the infection. In contrast, the protective antibody responses against malaria sporozoites induced by RTS,S depend on the neutralizing effect of antibodies present at the moment of sporozoite infection. This neutralization needs to occur swiftly, as sporozoites may reach hepatocytes as soon as 10 to 15 minutes after injection (17). After hepatocyte invasion, antibodies are no longer effective, and eventually the CSP antigen will no longer be expressed. Thus, any recall response that may occur after the infection will have no effect on the ongoing infection.

Most malariologists believe that a vaccine capable of inducing protective immunity against all the stages of parasite infection is most likely to have the highest impact on infection, morbidity, and transmission of malaria. In this regard, it is worth mentioning that studies in Africa indicate that children in areas of moderate transmission who were vaccinated and protected after immunization with RTS,S undergo a significant increase in rebound episodes of clinical malaria 3 to 6 years later, and this is likely due to a decreased immune response against blood stages observed in these individuals (18). This strongly suggests that combining anti-sporozoite and anti-blood stage vaccines may not be just desirable but perhaps critically needed. Consequently, anti-sporozoite vaccines like RTS,S are an important first step in the development of multi-stage vaccines that will one day become a powerful tool to help with malaria eradication.

## Version 1 01/04/2022

Electronic publication
